# Supplementary material for: Analysis of PPARγ Signaling Activity in Psoriasis
Source: Int J Mol Sci. 2021 Aug 10;22(16):8603. doi: 10.3390/ijms22168603 (PMC8395241; doi:10.3390/ijms22168603)
Supplement: Supplementary file 1 [file ijms-22-08603-s001.zip › Supplemental materials_Analysis of PPARg signaling activity in psoriasis/Pathway models/Models images and html files/Interleukin-17 and interleukin-22 signaling in psoriasis/191948.html]

ECM degradation


# Cell Process ECM degradation

|  |  |
| --- | --- |
| URN | urn:agi-aocellp:0000700 |
| Total Entities | 0 |
| Connectivity | 1402 |
| Name | ECM degradation |

---

|  |  |
| --- | --- |
| Pathway | Osteoarthritis |
|  | Skin Aging |
|  | Plasmin Effects in Inflammation |
|  | Bradykinin Effects in Inflammation |
|  | Leukotriene Effect on Vascular Endothelial Cell Response |
|  | Extracellular Matrix Turnover |
|  | Inflammatory Reaction in Acne Vulgaris |
|  | X-Linked Ichthyosis |
|  | Melanoma |
|  | Dedifferentiation and Metastatic Progression in Melanoma |
|  | Extracellular Matrix Degradation in Skin Aging |
|  | POMC Activation in Keratinocytes |
|  | Tubular Cell Dysfunction in Progressive Diabetic Nephropathy |
|  | Mesangial Cell Dysfunction in Diabetic Nephropathy |
|  | Bone Remodeling in Hyperthyroidism |
|  | SERPINA1 Associated Pulmonary Emphysema |
|  | TNF and IL1B Induce Metalloproteinase Synthesis in Osteoarthritis |
|  | TGFB2 Signaling Impairement in Osteoarthritis |
|  | Synovial Fibroblast Activation in Psoriatic Arthritis |
|  | Keratinocyte Activation in Psoriatic Arthritis |
|  | Synovial Fibroblast Activation by Citokines in Rheumatoid Arthritis |
|  | TLR2 Induced Synovial Fibroblast Activation in Rheumatoid Arthritis |
|  | Cathepsins Role in Periodontitis |
|  | Estrogen Effects on Pregnancy Gingivitis |
|  | Glioma Invasion Signaling |
|  | Androgen Receptor/Prostate Specific Antigen Signaling in Prostate Cancer |
|  | Prostate Cancer |
|  | Netrin-1 Signaling in Age-Related Macular Degeneration |
|  | Steroids Induced Cataract |
|  | PGF2-alpha Effect on Treatment |
|  | Alveolar Macrophages Dysfunction in COPD |
|  | Neutrophil Associated Lung Damage in Chronic Obstructive Pulmonary Disease |
|  | Alveolar Epithelial Cell Dysfunction in COPD |
|  | Mesangial Cell Dysfunction in Glomerulonephritis |
|  | Interstitial Fibroblasts in Pyelonephritis |
|  | Integrins in Cancer Cell Motility, Invasion and Survival |
|  | Epithelial to Mesenchymal Transition in Cancer: Overview |
|  | TGFB Family in Epithelial to Mesenchymal Transition in Cancer |
|  | Invadopodia Formation in Cancer Cells |
|  | WNT in Epithelial to Mesenchymal Transition in Cancer |
|  | 1\_Inflammation in keratinocytes in the pathology of acne |
|  | 2\_Interleukin-17 and interleukin-22 signaling in psoriasis |
|  | 1\_TNF and IL1B provoke ECM degradation in osteoarthritis |
|  | 3\_TGFB signaling provokes endochondral ossification with osteophyte formation |
|  | 3\_The role of interstitial fibroblasts in pyelonephritis |
|  | 3\_Mesangial cell dysfunction in glomerulonephritis |
|  | 1\_Cigarette smoke causes dysfunction and production of inflammatory mediators in alveolar epithelial cells |
|  | 3\_Role of alveolar macrophages in pulmonary tissue destruction in COPD |
|  | 2\_3\_Eosinophilia and neutrophilia in asthma: Neutrophil chemotaxis and activation |
|  | 1\_2\_Feedback relations between coagulation and fibrinolysis |
|  | 1\_SERPINA1-associated pulmonary emphysema |
|  | Interleukin-17 and interleukin-22 signaling in psoriasis |

---

|  |  |
| --- | --- |
| MedScan ID | 4160194 |

---

|  |  |
| --- | --- |
| Alias | degradation of the extracellular matrix |
|  | extracellular matrix catabolism |
|  | destruction of the extracellular matrix |
|  | extracellular matrix destruction |
|  | ECM catabolisms |
|  | extra-cellular matrix degradations |
|  | ECM catabolism |
|  | extracellular matrix destructions |
|  | extracellular matrix catabolisms |
|  | ECM breakdowns |
|  | extra-cellular matrix degradation |
|  | extracellular matrix breakdown |
|  | degradation of the extra-cellular matrix |
|  | ECM degradation |
|  | ECM degradations |
|  | breakdown of extracellular matrix |
|  | ECM breakdown |
|  | extracellular matrix breakdowns |
|  | catabolism of the extracellular matrix |
|  | degradation of ECM |
|  | extracellular matrix degradation |
|  | extracellular matrix degradations |
|  | extracellular proteolytic activity |

---

|  |  |
| --- | --- |
| Cell Localization | Extracellular |

---
